# Dosimetric comparison of Acuros XB with collapsed cone convolution/superposition and anisotropic analytic algorithm for stereotactic ablative radiotherapy of thoracic spinal metastases

**DOI:** 10.1120/jacmp.v16i4.5493

**Published:** 2015-07-08

**Authors:** Heming Zhen, Brian Hrycushko, Huichen Lee, Robert Timmerman, Arnold Pompoš, Strahinja Stojadinovic, Ryan Foster, Steve B. Jiang, Timothy Solberg, Xuejun Gu

**Affiliations:** ^1^ Department of Radiation Oncology UT Southwestern Medical Center Dallas TX; ^2^ Department of Radiation Oncology University of Pennsylvania Perelman Center for Advanced Medicine Philadelphia PA USA

**Keywords:** Acuros, heterogeneity correction, stereotactic, SBRT, spine radiotherapy

## Abstract

The aim of this study is to compare the recent Eclipse Acuros XB (AXB) dose calculation engine with the Pinnacle collapsed cone convolution/superposition (CCC) dose calculation algorithm and the Eclipse anisotropic analytic algorithm (AAA) for stereotactic ablative radiotherapy (SAbR) treatment planning of thoracic spinal (T‐spine) metastases using IMRT and VMAT delivery techniques. The three commissioned dose engines (CCC, AAA, and AXB) were validated with ion chamber and EBT2 film measurements utilizing a heterogeneous slab‐geometry water phantom and an anthropomorphic phantom. Step‐and‐shoot IMRT and VMAT treatment plans were developed and optimized for eight patients in Pinnacle, following our institutional SAbR protocol for spinal metastases. The CCC algorithm, with heterogeneity corrections, was used for dose calculations. These plans were then exported to Eclipse and recalculated using the AAA and AXB dose calculation algorithms. Various dosimetric parameters calculated with CCC and AAA were compared to that of the AXB calculations. In regions receiving above 50% of prescription dose, the calculated CCC mean dose is 3.1%–4.1% higher than that of AXB calculations for IMRT plans and 2.8%–3.5% higher for VMAT plans, while the calculated AAA mean dose is 1.5%–2.4% lower for IMRT and 1.2%–1.6% lower for VMAT. Statistically significant differences (p<0.05) were observed for most GTV and PTV indices between the CCC and AXB calculations for IMRT and VMAT, while differences between the AAA and AXB calculations were not statistically significant. For T‐spine SAbR treatment planning, the CCC calculations give a statistically significant overestimation of target dose compared to AXB. AAA underestimates target dose with no statistical significance compared to AXB. Further study is needed to determine the clinical impact of these findings.

PACS number: 87.55.D‐, 87.53.Ly

## I. INTRODUCTION

Stereotactic ablative radiotherapy (SAbR), also known as stereotactic body radiation therapy (SBRT), is an effective treatment option for patients with primary or metastatic malignancies. The rationale for SAbR of the spine is demonstrated in excellent pain control, bone marrow sparing in adjacent vertebrae, and a conventional, short treatment course and recovery time.^1^, ^2^, ^3^, ^4^, ^5^, ^6^ With ablative dose delivery, treatment efficacy is balanced with dose‐related toxicity to the spinal cord, requiring a sharp dose gradient outside the target region. Sophisticated treatment planning techniques, such as intensity‐modulation radiation therapy (IMRT) and volumetric‐modulated arc therapy (VMAT), are used to shape the dose in this fashion. High‐quality treatment plans rely upon accurate dose calculations. Boyer and Schultheiss^(7)^ reported that a 1% improvement in accuracy results in a 2% increase in the cure rate for early stage tumors. AAPM Report No. 85 states that a 5% change in dose may result in a 10% to 20% change in tumor control probability (TCP). Similarly, a 5% change in dose may result in a 20% to 30% change in complication rates of normal tissues.^(8)^ SAbR of the thoracic spine (T‐spine) requires photon beams to pass through tissues highly heterogeneous in composition and structure. A dose calculation engine accurately accounting for these heterogeneities would ultimately provide for a greater understanding of the radiation dose‐response relationships for tumors and normal tissues.

The collapsed cone convolution/superposition (CCC) algorithm in Pinnacle (Philips Medical Systems, Andover, MA) and the anisotropic analytical dose algorithm (AAA) in Eclipse (Varian Medical Systems, Palo Alto, CA) are commonly used heterogeneous dose calculation engines which perform adequately in most clinical applications.^(9)^ The CCC dose engine determines dose deposition by a three‐dimensional convolution/superposition of the Total Energy Released per unit Mass (TERMA) with a dose spread function. The TERMA is determined by projection of the beam energy fluence through the patient CT volume. The effects of changes in tissue composition on dose distribution are approximated by scaling the dose spread function by the radiological distance to account for the presence of heterogeneities with respect to scattered radiation. The superposition of the calculated dose distribution from all TERMA volumes determines the final 3D dose distribution in patient. The AAA algorithm is based on a pencil beam convolution/superposition technique. Heterogeneity corrections are accomplished longitudinally by radiological depth scaling along ray directions and laterally by combining a radiological distance scaling to exponential absorption functions. The heterogeneity corrections are approximate and have been shown to underestimate (or overestimate) dose at bone/air/tissue interfaces.^10^, ^11^, ^12^

The Acuros XB (AXB) (Varian Medical Systems) and AAA algorithms share the same photon beam source model,^(13)^ but differ in radiation transport and dose deposition throughout the patient volume. AXB, similar to the Monte Carlo algorithm, explicitly models the physical interaction of radiation in media and solves the Linear Boltzmann Transportation Equations (LBTE) to calculate the energy‐dependent fluence. It has been shown to agree well with Monte Carlo calculations and phantom measurements in heterogeneous media.^14^, ^15^, ^16^, ^17^, ^18^, ^19^, ^20^, ^21^, ^22^ The dose is obtained by multiplying the calculated fluence with a fluence‐to‐dose response function reported as dose‐to‐medium or dose‐to‐water. Bush et al.^(14)^ found that AXB algorithm's dose calculation in heterogeneous geometry agrees with Monte Carlo within 2.0% in regular lung material and within 2.9% in low‐density lung material, while difference between AAA and Monte Carlo was up to 10.2% and 17.5%, respectively. Han et al.^(15)^ performed TLD measurements in RPC thorax phantom and found that AXB calculation was within 0.4%–4.4% of the measured results. Fogliata et al.^(21)^ showed good agreement between Acuros XB and Monte Carlo, even in extreme cases of materials of very low density and for low energy and small fields. Dose calculations from AAA, AXB, CCC, and Monte Carlo have been compared for phantoms (homogenous and heterogeneous) and patients for a variety of treatment sites (e.g., breast, lung, nasopharyngeal carcinomas, and metallic hip implant^15^, ^16^, ^23^, ^24^, ^25^, ^26^, ^27^). This work is the first investigation on the dosimetric differences of intensity‐modulated and volumetric‐modulated arc treatment plans calculated with AXB, CCC, and AAA for SAbR of the T‐spine.

## II. MATERIALS AND METHODS

### A. Treatment planning systems commissioning and validation

Our Pinnacle (version 9.4) and Eclipse (version 10.0.8) treatment planning systems are commissioned for SAbR treatments on multiple disease sites, including head and neck, lung, spine, and liver. During TPSs commissioning, a composite set of beam data was collected from Varian TrueBeam linear accelerators equipped with Millenium 120 leaf MLC. Depth dose and beam profiles were measured with a Wellhofer water phantom (IBA Dosimetry, GmbH, Germany) using IBA CC13 (0.13 cm^3^ volume and 6 mm diameter), PTW 31014 (0.015 cm^3^ volume and 2 mm diameter; PTW, Freiburg, Germany) ion chambers and the Sun Nuclear Edge Detector SFD‐3G (0.0019 mm^3^ active detection volume and $0.8\times 0.8\;{\text{mm}}^{2}$ active detection area (Sun Nuclear Corporation, Melbourne, FL). Field sizes $\geq 4\times 4\;{\text{cm}}^{2}$ were scanned with CC13 and the smaller ones were scanned by Edge Detector and verified by PTW 31014. All measurements were made with a source‐to‐surface distance of 100 cm SSD. For Pinnacle TPS, beam data collection follows the Pinnacle^3^ Beam Data Collection Guide. Crossline and inline profiles were measured at four depths ($d_{\text{max}}$, 5, 10, and 20 cm) for each field size. Measured field sizes ranged from 1×1 up to $40\times 40\;{\text{cm}}^{2}$ formed by jaws and MLC. For Eclipse TPS, beam data collection follows Eclipse TPS reference guide, requiring open beams ranged from 2×2 up to $40\times 40\;{\text{cm}}^{2}$ formed by jaws at five depths ($d_{\text{max}}$, 5, 10, 20, and 30 cm). All scans were postprocessed according to the guidelines provided in AAPM Task Group 106.^(28)^ Output factors were measured at a depth of 10 cm in water using the CC13 ion chamber for field sizes $3\times 3\;{\text{cm}}^{2}$ and larger, and the Sun Nuclear Edge Detector for the 1×1 and $2\times 2\;{\text{cm}}^{2}$ field sizes. Absolute dose calibration was performed in accordance with the AAPM TG‐51 protocol. Following TPS commissioning guidelines,^29^, ^30^ beam models for Eclipse and Pinnacle were evaluated by direct comparison of the TPS beam model with the input measured profiles. Treatment plans of varying complexity, including single open beams shaped by multileaf collimator and IMRT and VMAT testing plans on existing patient CT scans, were created with both systems and delivered to homogenous solid water phantom for validation.

**Figure 1:**
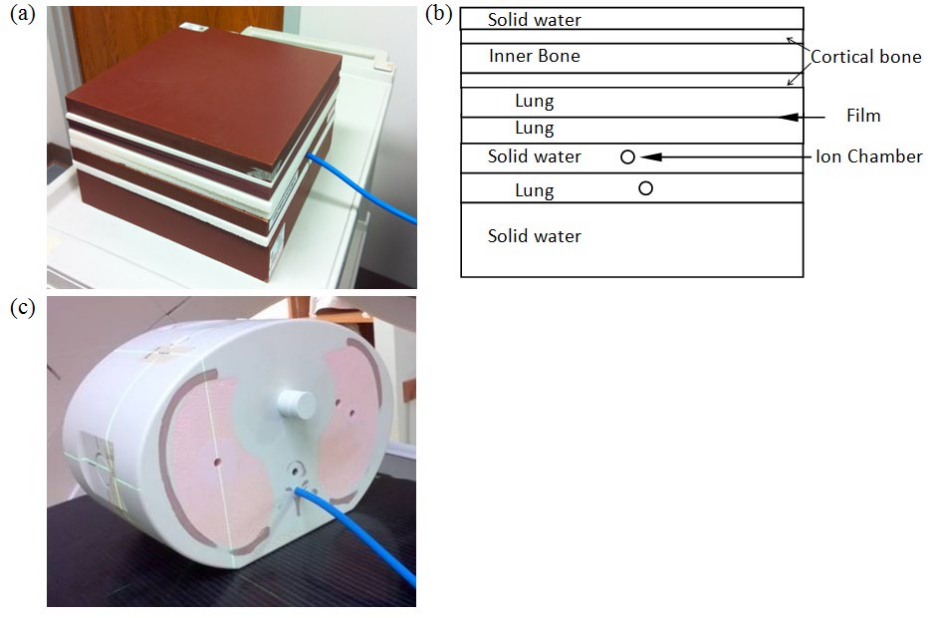
Heterogeneous slab‐geometry phantom (a) and (b) its schematic diagram for measurement setup; (c) the anthropomorphic phantom.

Specific TPS validation tests for this study were also conducted. Single field 6 MV open beams with field size $2\times 2\;{\text{cm}}^{2},3\times 3\;{\text{cm}}^{2}$, and $5\times 5\;{\text{cm}}^{2}$ were created and delivered on a heterogeneous slab‐geometry phantom (Gammex, Inc. Middleton, WI) composed of water, cortical bone, inner bone, and lung‐equivalent slabs to verify absolute and relative dose in heterogeneous media. Figures 1(a) and (b) show heterogeneous slab‐geometry phantom setup. Furthermore, an IMRT plan and a VMAT arc plan for T‐spine SBRT were created on the anthropomorphic thoracic phantom (Integrated Medical Technologies, Troy, NY) consisting of a solid water body with tissue‐equivalent lungs, spinal cord, vertebrae, and ribs. The anthropomorphic phantom, as shown in Fig. 1(c), has a special design in spine cord for holding PinPoint chamber (PTW), where absolute point dose was measured. All aforementioned absolute point doses were measured using a PTW 31014 ion chamber and relative planar dose distributions were measured using Gafchromic EBT3 (ISP Corporation, Wayne, NJ) film. Relative agreement between the TPS calculation and the measured dose distribution was assessed using gamma analysis.

### B. T‐spine SAbR treatment planning

Eight patients having T‐spine metastases were previously treated following our institutional SAbR spine protocol. Patients were immobilized in a supine position with a customized body vacuum bag within an Elekta stereotactic body frame (SBF) (Elekta Oncology Systems, Crawley, UK). CT images were acquired with a 2 mm slice thickness. MR images were acquired with the patient in the same position to aid in delineation of the target, spinal cord, and other organs at risk (OARs) when fused with CT images. Treatment plans using 6 MV photon beams were developed in Pinnacle v9.4. Thirteen to fifteen coplanar step‐and‐shoot beams were used for IMRT plans and one‐to‐two arcs were used for VMAT (Pinnacle SmartArc) plans. Dose is typically prescribed with 14 Gy given to at least 95% of the PTV and 20 Gy given to at least 90% of the GTV in 1 fraction and the prescription isodose line is around 70%–90%. IMRT and VMAT plans were optimized using the dose objectives shown in Table 1, which are based on OAR tolerance data, radiobiological models, and norms used in current clinic practice.^(31)^ All treatment plans met dosimetric endpoint constraints, as listed in Table 1. The dose to other OARs, such as lung, is far below the dosimetric constraints. Dose calculations in Pinnacle were performed using the CCC algorithm with heterogeneous corrections on a dose grid with $3.0\times 3.0\times 2.0\;{\text{mm}}^{3}$ resolution

The Pinnacle treatment plans contours, points of interest, beam arrangements, and dose distribution were exported to the Eclipse TPS. The imported plans were kept in Eclipse for dosimetric metrics readout and a copied plan was generated by recalculating dose using the AXB (version 10.0.28) and AAA (version 10.0.28) algorithms with the same monitor units and dose grid resolution. For Acuros dose calculation, dose reporting option was set to the default dose‐to‐medium, avoiding uncertainties when converting dose‐to‐medium to dose‐to‐water.^(32)^ The Eclipse system requires “Body” contours and only uses CT information enclosed within this region. “Body” contours enclosing the SBF were generated in Eclipse to ensure the two planning systems calculate dose on the same patient and frame geometry.

**Table 1:** Dose constraints used in inverse planning

*Structure*	*Parameters*	*Requirement*
GTV	$V_{100}$(Volume that receives ≥20 Gy)	≥90%
	CI^a^	<2.0
PTV	V100(Volume that receives ≥14 Gy)	≥95%
	V90(Volume that receives ≥12.6 Gy)	≥99%
	CI^a^	<1.3
Spinal Cord	$D_{\text{max}}$	<14Gy
	$D_{0.25\text{cc}}$	<10Gy
	$D_{0.5\text{cc}}$	<7Gy
Esophagus	$D_{\text{max}}$	<14.5Gy
	$D_{5\text{cc}}$	<11.9Gy

a ^a^ Conformity Index (CI) is defined as the ratio of the volume of the prescription isodose line over the volume of the GTV (PTV)

### C. Evaluation of dose distribution

The dose distributions calculated by the AAA and CCC dose calculation algorithms were compared with that of the AXB algorithm in terms of line profiles, voxel percentage dose differences, and shape of isodose lines. Three dimensional γ‐index distributions were calculated using a fast g‐Gamma engine^(33)^ with the AXB dose used as the reference dose. The voxel percentage dose difference is a local percentage difference, defined as $\frac{D_{eval}-D_{ref}}{D_{ref}}\times 100\%$ at each voxel, where $D_{\text{eval}}$ is evaluation dose calculated with CCC and AAA, and $D_{\text{ref}}$ denotes to reference dose calculated with AXB, while the gamma evaluation is relative to a global prescription dose (20 Gy). A 2% dose difference (global percentage difference) and 2 mm distance to agreement (2%/2 mm) was used as the gamma evaluation passing criteria. To provide greater detail into dose comparisons, the voxel percentage dose difference and gamma evaluations were performed in four subregions: $R_{2‐10}$ (2 Gy to 10 Gy), $R_{10‐14}$ (10 Gy to 14 Gy), $R_{14‐20}$ (14 Gy to 20 Gy), and $R_{20--}$ (above 20 Gy).

### D. Evaluation of critical dosimetric parameters

To investigate the potential clinical impact from differences in the dose calculations between AXB and AAA/CCC, dose‐volume analysis was performed for each target, spinal cord, and esophagus. Target (GTV and PTV) doses were compared in terms of the $D_{95}$ (dose covering 95% of the target volume), $D_{99}$ (dose covering 99% of the target volume), $D_{\text{mean}}$ (mean target dose), $V_{100}$ (percentage of the volume receiving 100% of the prescription dose 20 Gy), and $V_{90}$ (percentage of the volume receiving 90% of the prescription dose 20 Gy).

Clinically relevant dose metrics for the serial‐structured spinal cord and esophagus OARs were also compared. The $D_{\text{max}}$ (maximum dose), $D_{0.25\text{cc}}$ (minimum dose received by the maximally exposed 0.25 cc volume), and $D_{0.5\text{cc}}$ (minimum dose received by the maximally exposed 0.5 cc volume) were compared for the spinal cord. For the esophagus, the $D_{\text{max}}$ and the $D_{5\text{cc}}$ (minimum dose received by the maximally exposed 5 cc volume) are reported. Each metric was compared using a two‐tailed, paired Student's *t*‐test to test for statistically significant differences (p<0.05) between any two algorithms.

## III. RESULTS

### A. TPS validation on heterogeneous slab phantom and anthropomorphic phantom

For TPS validation with small field open beams, the absolute dose was measured with a PinPoint chamber in the Solid Water slab and the relative dose were measured by film located between two lung slabs (see Fig. 1(b)). The percentage difference between ionization chamber measurements and the calculated point doses for $2\times 2\;{\text{cm}}^{2},3\times 3\;{\text{cm}}^{2},5\times 5\;{\text{cm}}^{2}$ fields were 0.82%, 0.55%, and 0.22% for AXB dose calculation, 0.71%, 0.43%, and 0.20% for AAA dose calculation, and 0.93%, 0.75%, and 0.32% for CCC dose calculation, respectively. All film measurements had a 90% or higher gamma passing rate when using a 3%/3 mm passing criterion. The ionization chamber measurement within anthropomorphic thoracic phantom spinal cord inserts was compared to calculated point dose and the point dose measurement was within 3.0% of the AXB, AAA, and CCC calculations for IMRT and arc plans.

### B. Dose distribution comparison

**Figure 2:**
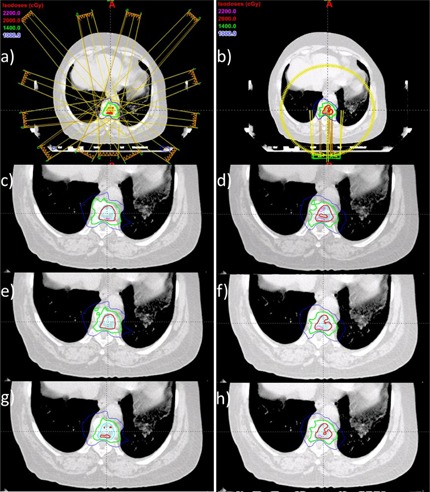
Subfigures in the first row show the beam configuration of (a) IMRT and (b) VMAT for a sample patient. Rows 2‐4 show the isodose distribution for different technique/algorithm combination as follows: (c) IMRT/CCC, (d) VMAT/CCC, (e) IMRT/AXB, (f) VMAT/AXB, (g) IMRT/AAA, and (h) VMAT/AAA.

**Figure 3:**
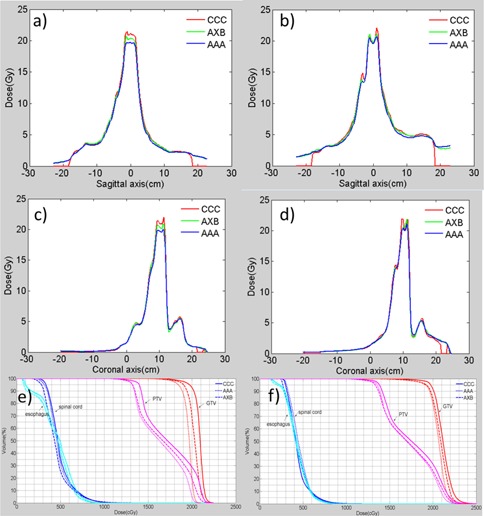
Left column illustrates (a) dose profiles along sagittal axis and (c) coronal axis, and (e) DVH for an IMRT plan; right column illustrates (b) dose profiles along sagittal axis and (d) coronal axis, and (f) DVH for a VMAT plan. Notice there are some zero segment on the CCC dose profile; this is due to the fact that pinnacle automatically assign a zero dose on air volume outside the body and frame.

Figure 2 shows a sample IMRT (left column) and VMAT plan (right column), where the first row shows the plan's beam arrangement, and the second to fourth rows show axial views of isodose distribution calculated using CCC, AXB, and AAA, respectively. In both IMRT and VMAT plans, the isodose lines show variations between different dose calculation engines. At medium‐ and high‐dose levels, the shapes of isodose lines calculated with CCC are quite different from the isodose curves calculated with AAA and AXB (e.g., the 14 Gy isodose line). Dose profiles passing through isocenter along the sagittal and the coronal axis are shown in Fig. 3. The dose profiles show CCC calculates a higher dose than AXB and AAA, and this is more pronounced in high‐dose regions.

**Figure 4:**
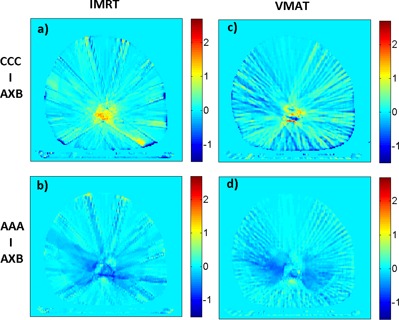
Percentage dose difference of a sample patient in an axial slice: (left) an IMRT plan (a) CCC versus AXB, (b) AAA versus AXB; and (right) a VMAT plan (c) CCC versus AXB, (d) AAA versus AXB.

Figure 4 shows an axial slice of a patient volume with voxel percentage dose differences mapped between the CCC/AAA and the AXB dose calculations. For both IMRT and VMAT plans, there are noticeable dose differences between the CCC/AAA and the AXB dose in the target region, with the CCC dose being higher than AXB, and the AAA dose being lower than AXB. The statistics of local percentage dose difference between the three dose engines are summarized in Table 2. The mean value gives the average voxel percentage dose difference of all six patients with AXB used as the reference dose. For both IMRT and VMAT plans, the CCC dose calculations are 3%–4% higher relative to AXB in the medium to high dose regions, while dose calculated AAA doses are approximately 1.5%–2.5% lower compared to AXB. In the low‐dose region, AAA shows small differences compared to AXB, while the CCC dose is approximately 2% higher than AXB.

**Table 2:** The statistics of local percentage dose difference and 3D gamma passing rate (2%/2 mm) of AAA and CCC compared to AXB within different dose regions for six patients IMRT and VMAT plans

			*AAA vs. AXB*				*CCC vs. AXB*			
			$R_{2‐10}$	$R_{10‐14}$	$R_{14‐20}$	$R_{20-}$	$R_{2‐10}$	$R_{10‐14}$	$R_{14‐20}$	$R_{20-}$
IMRT	%Diff	Mean	−0.7%	−1.5%	−1.5%	−2.4%	2.0%	4.1%	4.0%	3.1%
		STD	0.7%	0.8%	0.9%	1.2%	1.3%	0.3%	0.3%	0.8%
	γ	Mean	98%	99%	98%	70%	83%	96%	92%	87%
		STD	±1%	±3%	±3%	±28%	±6%	±4%	±8%	±15%
VMAT	%Diff	Mean	0.0%	−1.2%	−1.2%	−1.6%	2.3%	3.3%	3.5%	2.8%
		STD	1.9%	0.5%	0.4%	0.7%	2.7%	0.6%	0.8%	1.6%
	γ	Mean	100%	100%	99%	90%	88%	97%	97%	95%
		STD	0%	±1%	±2%	±9%	±10%	±2%	±3%	±4%

The three‐dimensional gamma passing rates using a 2%/2 mm passing criterion are tabulated in Table 2. Here we should point out that the tabulated gamma passing rate doesn't include regions where CCC assign dose to 0 Gy on air volume outside body and frame. AAA vs. AXB has a higher gamma passing rate in all regions except for $R_{20-‐}$, indicating that the difference between AAA and AXB is smaller than that between CCC and AXB in these regions. It is interesting to see that the gamma passing rate of AAA/AXB is lower than CCC/AXB in the highest dose region, namely $R_{20-‐}$, which contradicts the observation of the percentage dose differences.

### C. Clinically relevant DVH parameters comparison

The dose difference in the high‐dose region is apparent in the shift of the GTV and PTV DVH curves in Figs. 3(e) and (f). For both IMRT and VMAT plans, target DVH differences between AXB and CCC calculations are relatively large compared to the agreement between AXB and AAA. AXB on average computes a lower spinal cord dose compared to AAA and CCC; however, such difference is not statistically significant.

Table 3 summarizes the mean and standard deviation of selected dosimetric parameters to targets and OAR. Two tailed p‐values in this table are obtained through paired *t*‐test on selected dose parameters. For both IMRT and VMAT plans, the GTV dose ($D_{\text{mean}},D_{95}$, and $D_{99}$) calculated by CCC is 2.2%–4.7% higher than AXB, and AAA is approximately 1%–2% lower than AXB. The PTV dose ($D_{\text{mean}},D_{95}$, and $D_{99}$) has a similar pattern. Based on the p‐value from the paired *t*‐test, the difference in the target dose ($D_{\text{mean}},D_{95}$, and $D_{99}$) between CCC and AXB is statistically significant for both IMRT plans (four out of six p‐values are less than 0.01) and VMAT plans (six out of six p‐values are less than 0.01). In contrast, such differences between AAA and AXB are generally not statistically significant. The differences between $V_{x}$ ($V_{100}$ and $V_{90}$) values follow a similar trend, but are all statistically insignificant. However $V_{x}$ values are too sensitive to small DVH shifts, resulting in very large uncertainties.

**Table 3:** Comparison of dosimetric parameters between three dose algorithms in IMRT and VMAT plans

						*p‐value*		
			*Mean(STD)*					
	*Structure*	*Parameter*	*CCC*	*AXB*	*AAA*	*CCC‐AXB*	*CCC‐AAA*	*AXB‐AAA*
IMRT	GTV	$V_{100}$	93% (±3%)	80% (±20%)	64% (±25%)	0.093	0.035	0.109
		$D_{\text{mean}}$ (Gy)	21.8 (±0.9)	21.3 (±1.0)	21.0 (±1.0)	0.030	<0.01	<0.01
		$D_{95}$ (Gy)	19.8 (±0.4)	19.2 (±0.6)	19.0 (±0.5)	<0.01	0.084	<0.01
		$D_{99}$ (Gy)	18.0 (±1.0)	17.7 (±1.4)	17.6 (±1.0)	<0.01	<0.01	0.526
	PTV	$V_{100}$	97% (±2%)	92% (±7%)	92% (±8%)	0.124	0.136	0.136
		$V_{90}$	99% (±1%)	98% (±1%)	99% (±1%)	0.390	0.227	0.068
		$D_{\text{mean}}$ (Gy)	17.7 (±1.5)	17.1 (±1.3)	17.0 (±1.3)	0.048	<0.01	0.640
		$D_{95}$ (Gy)	15.0 (±1.9)	14.3 (±1.8)	14.4 (±1.8)	<0.01	<0.01	0.159
		$D_{99}$ (Gy)	13.3 (±1.9)	12.6 (±1.9)	12.9 (±1.7)	<0.01	<0.01	0.059
	Cord	$D_{\text{max}}$ (Gy)	1.0 (±1.2)	9.7 (±1.3)	9.9 (±1.2)	0.377	0.012	<0.01
		$D_{0.25\text{cc}}$ (Gy)	8.1 (±0.9)	7.3 (±1.1)	7.6 (±1.0)	0.119	<0.01	0.010
		$D_{0.5\text{cc}}$ (Gy)	6.6 (±0.9)	5.8 (±1.0)	6.2 (±0.9)	0.108	<0.01	<0.01
	Esophagus	$D_{\text{max}}$ (Gy)	12.7 (±3.1)	12.3 (±3.1)	12.3 (±3.1)	<0.01	<0.01	0.050
		$D_{5\text{cc}}$ (Gy)	3.2 (±2.2)	2.9 (±2.0)	3.0 (±2.1)	0.777	0.037	0.017
	Rx Isodose line		84% (±5%)	87% (±5%)	91% (±5%)	<0.01	<0.01	<0.01
VMAT	GTV	$V_{100}$	93% (±2%)	82% (±15%)	79% (±15%)	0.067	0.027	0.069
		$D_{\text{mean}}$	21.6 (±0.7)	21.1 (±0.6)	20.9 (±0.6)	<0.01	<0.01	0.218
		$D_{95}$ (Gy)	19.8 (±0.3)	19.2 (±0.4)	19.1 (±0.3)	<0.01	<0.01	0.121
		$D_{99}$ (Gy)	18.3 (±0.8)	17.6 (±0.9)	17.6 (±0.8)	<0.01	0.014	0.942
	PTV	$V_{100}$	96% (±4%)	92% (±7%)	92% (±6%)	0.119	0.012	0.219
		$V_{90}$	99% (±2%)	98% (±3%)	98% (±3%)	0.468	0.060	0.019
		$D_{\text{mean}}$ (Gy)	17.6 (±1.8)	17.1 (±1.7)	17.1 (±1.7)	<0.01	<0.01	0.991
		$D_{95}$ (Gy)	14.7 (±2.0)	14.1 (±2.0)	14.2 (±1.9)	<0.01	<0.01	0.027
		$D_{99}$ (Gy)	13.0 (±2.1)	12.3 (±2.1)	12.5 (±2.0)	<0.01	<0.01	<0.01
	Cord	$D_{\text{max}}$ (Gy)	10.4 (±1.9)	10.2 (±2.2)	10.4 (±2.2)	0.096	0.013	0.098
		$D_{0.25\text{cc}}$ (Gy)	8.1 (±1.7)	7.9 (±1.9)	8.0 (±1.9)	0.364	0.801	<0.01
		$D_{0.5\text{cc}}$ (Gy)	6.6 (±1.7)	6.4 (±2.0)	6.6 (±1.9)	0.806	0.867	<0.01
	Esophagus	$D_{\text{max}}$ (Gy)	12.7 (±3.3)	12.3 (±3.1)	12.2 (±3.1)	0.853	<0.01	0.100
		$D_{5\text{cc}}$ (Gy)	3.2 (±2.7)	3.0 (±2.7)	3.6 (±2.6)	0.922	0.523	<0.01
	Rx Isodose Line		83% (±5%)	86% (±6%)	87% (±5%)	<0.01	<0.01	<0.01

Table 3 also lists Rx isodose level (the ratio of the prescription dose to the maximum dose) determined by the three different dose engines. As expected, CCC yields the lowest Rx isodose line and AAA gives the highest. For spinal cord, the difference between the mean values of evaluated dose parameters over six IMRT plans and three algorithms is very small, and no statistically significance is observed in either IMRT or VMAT plans. The mean value of the maximum esophagus dose over six patient plans shows CCC gives highest value among three algorithms and AAA the lowest, while the dose difference on D5cc is minor for three algorithms.

## IV. DISCUSSION

Accurate dose calculations in SAbR treatment planning for T‐spine metastases are challenged by the increased atomic number in bone and a lack of lateral scattering equilibrium for small fields in the lungs. Based on previous validations with MC calculations, this work assumed AXB calculates dose accurately in this highly heterogeneous region and was taken as the standard from which to compare the AAA and CCC algorithms. The AAA algorithm was shown to underestimate, while the CCC algorithm overestimates, the dose inside targets (including the vertebral body and the bone marrow) compared to the AXB calculations. This is in agreement with the results of Carrasco et al.^(34)^ where five dose calculation algorithms of Helax‐TMS and Varian‐Eclipse were compared with MC calculations and TLD measurements in a slab phantom geometry using a cortical bone‐equivalent heterogeneity. In that study, CCC was shown to overestimate the dose inside the bone‐equivalent material by 3%−5% for 6 MV beams. The overestimation in dose calculations by the CCC algorithm can be explained by improper scaling of the water‐derived dose deposition kernels due to the greater scattering power from the higher atomic number of bone. This finding would indicate that, if CCC is used clinically for thoracic spine SAbR treatment planning, there will be a statistically significant 3%–5% underdose to the target. In theory, this reduces the tumor control probability and makes it difficult to accurately relate an absorbed dose index to response. Demonstrating this in a clinical setting would require a large patient population treated by plans using each algorithm.

Despite the differences within the targets between algorithms, it is reassuring that, when comparing CCC/AAA‐calculated dose to AXB with respect to dosimetric indices for the spinal cord and esophagus, no significant differences were observed. For spine SABR, the highest planning priorities are for spinal cord avoidance, resulting in consequential dose spilling anteriorly (near the esophagus) and laterally. Much of the esophagus is located at a lung/soft tissue interface, and the $D_{5\text{cc}}$ shows both AAA and CCC overestimate the dose. This is similar to observations in lung tumor cases, where kernel‐based models often overestimate the dose due to its incapability to model the electron‐photon coupled transport across the interface to account for forward/backward scattered photons and loss of electronic equilibrium.^11^, ^34^ Dose‐to‐medium was chosen to be the dose reporting methods in this case. The difference between dose‐to‐water and dose‐to‐medium calculation is that dose‐to‐water is dose‐to‐medium multiplied by the stopping power ratio between water and medium. Such difference is of essential importance for dose in bone. Fogliata et al.^(35)^ compared the two types of AXB calculation with AAA in VMAT treatment plans for leg sarcoma cases and found that dose‐to‐water scheme on average overestimate dose in bone by ∼5% compared to dose‐to‐medium and AAA. In theory, the dose‐to‐medium calculation better reflects the physical cross sections of the photon and electron interactions with matter, and has shown to have better agreement with conventional “rescaled‐to‐water” calculation (AAA and CCC).^(21)^

Another important factor effecting calculated dose distribution is dose calculation grid. Previous research studies^(36)^ have shown that the size of dose grid has impact on cord maximum dose and target coverage. In this study, we uniformly adopted a $3.0\times 3.0\times 2.0\;{\text{mm}}^{3}$ dose grid for dose calculation. The choice of such dose grid is based on our clinical protocol which balances between computational cost and clinical impact. And the uniform dose grid across all dose calculation engines eliminates the dose distribution variation caused by dose calculation grid. Though dose grid impact is an important concern from a clinical standpoint, it is beyond the scope of this study to evaluate various dose grids.

It is interesting to note that, despite the larger difference between CCC and AXB dose calculation in high‐dose regions ($R_{20-‐}$), the corresponding gamma passing rate in that region is actually better for the CCC vs. AXB comparison than for the AAA vs. AXB comparison. In this case, gamma passing rates are very sensitive to which dose map was assigned as the reference dataset. In the high‐dose region of Fig. 3, the CCC dose is higher than that of AXB, which is subsequently higher than AAA. Therefore, when calculating gamma between CCC and AXB, if AXB dose is chosen as a reference (which is the case in our study), it is much easier to satisfy the “distance‐to‐agreement” aspect than if CCC is chosen as a reference (in which case it's virtually impossible to find a distance‐to‐agreement match in the high‐dose region). When calculating gamma values between AAA and AXB, the opposite is true because the AAA dose is lower than the AXB dose in the high‐dose region. This essentially gives CCC an advantage for gamma evaluation when using AXB as the reference dataset.

## IV. CONCLUSIONS

This study evaluated the calculated dose differences between CCC and AAA dose engines with that of the AXB engine for T‐spine SAbR. By keeping the same prescription dose, CCC will overestimate the target dose compared to AXB, which ultimately leads to an underdose to the target of 3%–5%. AAA was shown to underestimate the target dose compared to AXB, although not statistically significant, which would lead to an overdose to the target of 0.5%–2%. However, uncertainties in application of the CCC and AAA regarding dose to the spinal cord and esophagus appear to be less. By not accounting for these differences in CCC or AAA dose calculations for SAbR treatment planning, the local control probability of thoracic spinal metastases could be modestly affected.
